# Comparison of periodontal status and salivary IL-15 and -18 levels in cigarette-smokers and individuals using electronic nicotine delivery systems

**DOI:** 10.1186/s12903-022-02700-6

**Published:** 2022-12-30

**Authors:** Dena Ali, Isaac Kuyunov, Jagan Kumar Baskaradoss, Toshinari Mikami

**Affiliations:** 1 Department of General Dental Practice, Kuwait University, Safat, P. O. Box 24923, 13110 Kuwait City, Kuwait; 2 Specialist in Prosthodontics, Dental Prosthodontics of Rochester, Rochester, NY 14618 USA; 3 Department of Developmental and Preventive Sciences, Kuwait University, Kuwait City, Kuwait; 4 Pax Creation Medical Lab, Morioka, Japan; 5 Department of Oral Pathology, Oral Lab Central College of Stomatology, China Medical University, Shenyang, China

**Keywords:** Alveolar bone loss, Clinical attachment loss, Electronic cigarette, Periodontitis, Plaque index, Smoking

## Abstract

**Background:**

It is hypothesized that periodontal status is compromised and whole salivary (WS) interleukin (IL)-15 and IL-18 levels are higher among cigarette-smokers and electronic-nicotine-delivery-systems (ENDS)-users than never-smokers. The aim of the present case–control study was to compare the periodontal status and WS IL-15 and -18 levels among cigarette-smokers, ENDS-users and controls (never-smokers).

**Methods:**

Participants were divided into 4 groups as follows: Group-1:Current cigarette-smokers; Group-2:ENDS-users; Group-3:Never-smokers with periodontitis; and Group-4: Never-smokers without periodontitis. Demographic data was collected and plaque index (PI), gingival index (GI), probing-depth (PD), clinical attachment-loss (AL), and marginal bone loss (MBL) were measured. Number of missing teeth were recorded and WS IL-15 and IL-18 levels were determined. Group-comparisons were done and *P* < 0.01 was selected as an indicator of statistical analysis.

**Results:**

Nineteen, 18, 19 and 19 individuals were enrolled in groups 1, 2, 3 and 4, respectively. Scores of PI, clinical AL, PD, and number of missing-teeth were elevated in groups 1(*P* < 0.001), 2 (*P* < 0.001) and 3 (*P* < 0.001) than -4. Scores of PI, clinical AL, PD, MBL and missing teeth were comparable among patients in groups 1, 2 and 3. Levels of IL-15 and IL-18 were elevated in groups 1 (*P* < 0.001) and 2 (*P* < 0.001) than groups 3 and 4. The levels of IL-15 and -18 were higher in Group-3 than in Group-4 (*P* < 0.001).

**Conclusion:**

Clinically, cigarette-smokers and never-smokers demonstrate similar periodontal statuses; however, WS immunoinflammatory biomarkers (IL-15 and -18) are elevated in these individuals than non-smokers.

## Introduction

Sufficient scientific evidence confirms that cigarette-smoking is associated with serious health including oral and systemic inflammatory conditions and malignancy [1–3]. Tobacco-smokers are usually reluctant to quit the habit as nicotine is addictive and its withdrawal elicits symptoms which encompass anxiety, psychological stress, and metabolic imbalances [4–9]. Nevertheless, those who are determined reduce or quit smoking rely on nicotine replacement therapies such as nicotine gums, lozenges, patches, and electronic nicotine delivery systems (ENDS) to replenish their bodily nicotine dependence and minimize the risk of nicotine withdrawal symptoms. According to a report published by the World Health Organization, nearly 35 million individuals vape or use ENDS throughout the globe; and these number are rapidly growing [10]. Ominously, this report also projected that by the year 2023, the vaping industry could make ENDS-sales reaching up to 40 billion United States dollars, which is nearly double of ENDS- sales in the year 2019 [10]. In 2013, the United States Food and Drug Administration authorized the commercial sale of ENDS as potential substitutes for quitting traditional tobacco smoking [11]. Although the precise prevalence of smoking and vaping among adult residents in Kuwait remains unclear, results from a recent cross-sectional study [12] on 1565 adolescents from Kuwait showed that habitual cigarette-smoking and vaping was reported by nearly 25% and 26% of the study population. ENDS users often perceive that vaping is a relatively less harmful alternate to traditional tobacco-smoking; however, scientific evidence has shown that habitual use of ENDS is a risk-factor of respiratory, cardiovascular, and neurological diseases [13–15].

From a periodontal perspective, it has been reported that plaque index (PI), clinical attachment loss (AL), and probing depth (PD) and marginal bone loss are higher in ENDS users than controls (individuals that have never used tobacco in any form) [16, 17]. Similarly, laboratory-based investigations have also shown that the immunoinflammatory biomarkers of inflammation such as receptor activator of NF-kappa B ligand, interleukin (IL)-1β, and tumor necrosis factor-alpha are expressed in raised concentrations in the oral fluids (such as unstimulated whole saliva [UWS] and gingival crevicular fluid [GCF]) of nicotinic-product users than controls [18, 19]. The IL-15 is a 14 to15 kDa glycoprotein and has a broad range of biological functions such as development of immune responses to microbial invaders and parasites by modulating immune cells [20]. Likewise, IL-18, is regulator of acquired as well as innate immunity. Acar et al. [21] assessed levels of IL-15 in the GCF of patients with periodontitis and controls. The outcome showed significantly higher levels of IL-15 in the GCF compared with controls [21]. Results from another study showed that serum levels of IL-18 are higher in the serum of patients with periodontitis than controls. However, whole salivary (WS) IL-15 and -18 levels in cigarette-smokers and ENDS users have not yet been investigated.

It is hypothesized that periodontal status is poorer, and WS IL-15 and -18 levels are higher among cigarette-smokers and ENDS-users compared with non-smokers. The aim was to compare periodontal condition and WS IL-15 and -18 levels among cigarette-smokers, ENDS-users and controls.

## Methods

### Patient eligibility criteria

Self-reported current cigarette-smokers, ENDS-users and never-smokers (controls) were included. Cigarette-smokers were defined as individuals who were currently smoking and had smoked at least 100 cigarettes during their life-time [22, 23]. Current ENDS-users were defined as individuals who had used ENDS at least once in the past 30 days [23]. Never-smokers were defined as volunteers who have never consumed combustible or non-combustible nicotinic products in any form [23, 24]. The definition of periodontitis was based on the consensus report by Papapanou et al. [25]. Dual-smoking, alcoholism, and self-reported systemic diseases (such as cardiovascular conditions, diabetes mellitus and cancer) were excluded. Patients that had used medications like antibiotics, bisphosphonates, and probiotics within three months were not sought. Moreover, third molars, nursing and pregnant females and patient undergoing cancer treatment were also excluded.

### Study groups

Participants were categorized into 4 groups as follows: Group-1: Current cigarette-smokers; Group-2: Current ENDS-users; Group-3: Never-smokers with periodontitis; and Group-4: Never-smokers without periodontitis.

### Questionnaire

A questionnaire was used to collect demographics and domestic oral health care data. Information was also collected on cigarette smoking duration and the number of cigarettes/packs smoked daily, duration and daily frequency of vaping, number of puffs inhaled during each session of ENDS use, and nicotine concentration in the liquid of electronic cigarettes (e-liquids). The questionnaire was administered by a trained investigator.

### Periodontal parameters assessed

PI [26], gingival index (GI) [27], clinical AL [28]; and PD [28] were rated. Bitewing radiographs were taken and MBL was examined on radiographs using a software program (Planmeca Romexis 2.2.7R software (Planmeca, Helsinki, Finland) as described elsewhere [29]. Numbers of missing teeth were also counted and recorded.

### Collection of unstimulated whole saliva and assessment of IL-15 and -18 levels

The UWS samples were non-invasively collected as described in a previous study [30]. In summary, UWS was collected with patients being in a fasting state. early morning hours with the participants being in a fasting state. The individuals were comfortably seated on a chair and requested to allow saliva to accumulate in the mouth for 5 continuous minutes. Patients were advised to refrain from swallowing and jaw movements. After 5-min, the participants drooled the UWS into a disposable plastic funnel that was attached to a gauged disposable measuring cylinder. The unstimulated whole salivary flow rate (UWSFR) was recorded and supernatant was stored at -80^o^ C. All UWS samples were collected by one trained and calibrated investigator (Kappa 0.88). The IL-15 (abcam, Human IL-15 ELISA Kit [ab218266], MA 02,139–1517 USA) and IL-18 (ELISA MAX™ Deluxe Set Human IL-18, R&D Systems Inc, USA). All kits were used according to the manufacturers’ instructions; and absorbance was read at 450 nm using a microplate reader (Multiskan GO, Thermo Scientifc, Finland). The results were presented as the picogram per milliliter (pg/mL).

### Blinding

Investigators that collected UWS samples and assessed the whole salivary IL-15 and -18 levels and periodontal status were blinded to the study groups. The statistician was also blinded to the group-definitions when the data was quantitatively evaluated.

### Statistical analysis and sample-size estimation

Statistical analyses were done using a software program (SPSS V. 22, Chicago, IL., USA). The Kolmogorov–Smirnov-test determined data normality. The clinicoradiographic and immunological parameters were compared among patients in groups 1, 2, 3 and 4 using the analysis of variance and Bonferroni Post-hoc adjustment tests. Correlation of IL-15 and -18 with clinical AL and PD was assessed using multiple logistic regression. Probability-values, which were less than 0.01 were nominated as being significant. Power and sample size analyses were determined using a computer software (nQuery Advisor 6.0, Statistical Solutions, Saugas, MA., USA) with an alpha and effect size of 0.01 and 0.3, respectively. It was appraised that with the addition of 18 individuals/group will be required to get an 80% power and detect a 1 mm difference in clinical AL and PD.

## Results

### Participants and their demographics

Nineteen, 18, 19 and 19 individuals were included in groups 1, 2, 3 and 4, respectively. In groups 1, 2, 3 and 4, 15, 12, 13 and 14 participants were male. The mean ages of participants in groups 1, 2, 3 and 4 were 52.6 ± 6.1, 49.5 ± 2.3, 50.7 ± 2.2 and 48.1 ± 1.3 years, respectively. In Group-1, the people had a smoking-history of 24.3 ± 0.7 packyears and in Group-2, individuals were vaping for 12.5 ± 0.8 years, respectively. In Group-2, the patients used ENDS 25.1 ± 3.5 times daily with 6.6 ± 0.7 puffs per session. In Group-2, all participants using ENDS used nicotine containing e-liquids and the mean concentration of nicotine in the e-liquids was 14.6 ± 4.05 mg/ml (Table 1). None of the individuals in groups 1, 2 and 3 remembered when they had last visited a dentist or a dental hygienist. In Group-4, 14 individuals (73.7%) reported to have visited their dental hygienist 7.7 ± 0.5 months ago. Toothbrushing twice daily was more often reported by participants in Group-4 (84.2%) compared with patients in groups 1 (31.6%), 2 (22.2%) and 3 (26.3%).

**Table 1:** Demographic characteristics of the groups

Parameter	Group-1	Group-2	Group-3	Group-4
Patients (n)	19	18	19	19
Gender (male: female)	15: 4	12: 6	13: 6	14: 5
Age in years (mean ± SD)	52.6 ± 6.1 years	49.5 ± 2.3 years	50.7 ± 2.2 years	48.1 ± 1.3 years
Duration of smoking (pack-years)	24.3 ± 0.7 pack years	NA	NA	NA
Duration of vaping (in years)	NA	12.5 ± 0.8 years	NA	NA
Daily frequency of vaping	NA	25.1 ± 3.5 times daily	NA	NA
Number of puffs inhaled with each vaping session	NA	6.6 ± 0.7 puffs per session	NA	NA
Nicotine concentration (mg/ml)	NA	14.6 ± 4.05 mg/ml	NA	NA

Group-1: Cigarette-smokers; Group-2: ENDS-users; Group-3: Never-smokers with periodontitis; and Group-4: Never-smokers without periodontitis; NA: Not applicable

### Periodontal parameters

Scores of PI, clinical AL, PD, mesial and distal MBL and number of missing teeth were significantly higher among patients in groups 1 (*P* < 0.001), 2 (*P* < 0.001) and 3 (*P* < 0.001) compared with Group-4. Scores of PI, clinical AL, PD, mesial and distal MBL and numbers of missing teeth were comparable among patients in groups 1, 2 and 3 (Table 2).

**Table 2:** Clinical and radiologic periodontal status

Parameter	Group-1	Group-2	Group-3	Group-4
Plaque index	3.1 ± 0.2*	2.5 ± 0.2*	2.3 ± 0.2*	0.3 ± 0.05
Gingival index	0.9 ± 0.04†	1.05 ± 0.03†	3.3 ± 0.05	0.5 ± 0.004^†^
Clinical attachment loss	8.4 ± 0.5 mm*	7.1 ± 0.4 mm*	7.8 ± 0.3 mm*	0.2 ± 0.003
Probing depth	6.5 ± 0.2 mm*	5.7 ± 0.2 mm*	6.1 ± 0.4 mm*	1.2 ± 0.06 mm
Marginal bone loss (mesial)	6.2 ± 0.7 mm*	5.8 ± 0.2 mm*	5.7 ± 0.2 mm*	0.4 ± 0.004 mm
Marginal bone loss (distal)	6.3 ± 0.6 mm*	5.5 ± 0.2 mm*	5.8 ± 0.3 mm*	0.3 ± 0.004 mm
Missing teeth	16.6 ± 2.3 teeth *	10.6 ± 1.2 teeth*	12.2 ± 1.5 teeth*	2.2 ± 0.5 teeth

*Compared with Group-4 (*P* < 0.001)

†Compared with Group-3 (*P* < 0.001)

Group-1: Cigarette-smokers; Group-2: ENDS-users; Group-3: Never-smokers with periodontitis; and Group-4: Never-smokers without periodontitis; NA: Not applicable

### Unstimulated whole salivary flow rate and whole salivary IL-15 and -18 levels

There was no difference in the UWSFR among individuals in groups 1, 2, 3 and 4. Levels of IL-15 and -18 were significantly higher among patients in groups 1 (*P* < 0.001) and 2 (*P* < 0.001) compared with those in groups 3 and 4. Levels of IL-15 and -18 were significantly higher among patients in Group-3 than Group-4 (*P* < 0.001). There was no difference in whole salivary IL-15 and -18 levels among individuals in groups 1 and 2 (Table 3). There was no difference in whole salivary IL-15 and -18 levels among males and females in all groups (data not shown).

**Table 3:** Unstimulated whole salivary flow rate and whole salivary IL-15 and -18 levels

Parameter	Group-1	Group-2	Group-3	Group-4
UWSFR (ml/min)	0.31 ± 0.05 ml/min	0.32 ± 0.02 ml/min	0.32 ± 0.04 ml/min	0.32 ± 0.05 ml/min
Interleukin-15 (pg/ml)	189.5 ± 26.7 pg/ml*†	174.7 ± 19.2 pg/ml*†	91.2 ± 14.3 pg/ml†	4.1 ± 0.2 pg/ml
Interleukin-18 (pg/ml)	2869.8 ± 285.6 pg/ml*†	2793.1 ± 196.4 pg/ml*†	1537.9 ± 101.8 pg/ml†	100.3 ± 2.5 pg/ml

*Compared with Group-3 (*P* < 0.001)

†Compared with Group-4 (*P* < 0.001)

Group-1: Cigarette-smokers; Group-2: ENDS-users; Group-3: Never-smokers with periodontitis; and Group-4: Never-smokers without periodontitis; NA: Not applicable

### Correlation between periodontal parameters, demographics, and salivary IL-15 and -18 levels

**Fig. 1:**
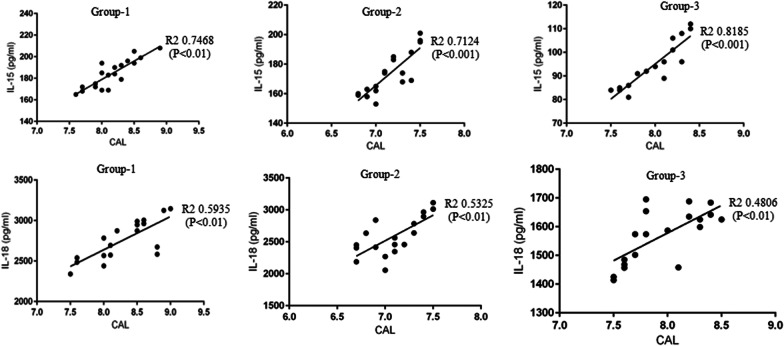
Correlation between clinical attachment loss and whole salivary IL-15 and -18 levels in the study groups

Figure 1 shows a statistically significant correlation between increasing clinical AL and WS IL-15 and -18 levels in groups 1, 2 and 3. This trend was not evident in Group-4 (data not shown). Logistic regression analysis showed no correlation between age, gender, smoking and vaping history, number of puffs vaped daily and nicotine concentration in e-liquids and WS IL-15 and -18 levels (data not shown).

## Discussion

Based upon the hypothesis, authors of the present study expected that scores of PI, PD, clinical AL and MBL would be significantly higher among patients in groups 1 and 2 (cigarette-smokers and ENDS-users, respectively) compared with never-smokers with periodontitis (Group-3). One reasoning for this is that smoking increases oxidative stress in periodontal tissues by increasing the interaction between advanced glycation endproducts and their receptors [31]. Interestingly, the present clinical results showed comparable periodontal scores in groups 1, 2 and 3. This suggests that the severity of periodontitis is independent of smoking status and if routine oral hygiene maintenance (brushing and flossing) are inappropriately executed, then never-smokers can display periodontal destruction that is similar to that observed in cigarette-smokers. The only clinical parameter that significantly varied among patients in groups -1,2 and 3 was GI, which was significantly higher among patients in Group-3 compared with groups 1 and 2 (Table 2). It has been reported that nicotine has a vasoconstrictive effect on gingival blood vessels [32, 33]; and this factor seems to explain the significantly lower cores of GI in groups 1 and 2 compared with Group-3. It is also noteworthy that GI scores of patients in groups 1 and 2 were comparable with those in Group-4. Since gingival bleeding is a clinical indicator of ongoing periodontal disease, it is speculated that tobacco-smokers and ENDS-users may remain unaware of the ongoing periodontal inflammatory process possibly due to the vasoconstrictive effect of nicotine on gingival vasculature. Despite the fact that the clinical presentation of periodontitis was similar among patients in groups 1, 2 and 3, it is noteworthy that WS IL-15 and -18 levels were nearly twice as high in groups -1 and -2 compared with Group-3. Moreover, there was no difference in the whole salivary IL-15 and -18 levels among cigarette-smokers and ENDs-users. These outcomes reflect that even though clinical and radiographic periodontal inflammatory parameters may be similar among nicotinic product users and never-smokers with periodontitis, the immunoinflammatory response on a molecular level is markedly raised in nicotinic product users. Our clinical and laboratory-based results also support previous studies [16, 34], which reported that vaping is not a “safe” or “less dangerous” alternate to conventional cigarette-smoking as periodontal destruction; and WS IL-15 and -18 levels were comparable among cigarette-smokers and ENDS-users (groups 1 and 2, respectively). Since usage of ENDS is by no means a “safe” replacement to traditional tobacco smoking, there is an urgent need to (a) educate the masses that both vaping is not “less harmful” to health than smoking; (b) monitor all forms of nicotine product use among all age groups; and (c) implement firm anti-tobacco and anti-vaping protocols.

Results based on logistic regression models showed no statistically significant correlation between age, gender, and concentration of nicotine inhaled, WS IL-15 and -18 levels. Relationship between nicotine concentration and clinical and laboratory-based parameters could only be assessed for patients in Group-2 as none of the patients in Group-1 (cigarette-smokers) were aware of the amount of nicotine present in each cigarette they smoked. However, individuals using ENDS were vaping commercially available e-liquids with nicotine concentrations ranging between 10 and 20 mg/ml. it has been reported that nicotine concentrations in e-liquids can range from 0 mg/ml (nicotine-free) to as high as 50 mg/ml nicotine [35]. It is therefore hypothesized that individuals vaping e-liquids containing higher nicotine concentrations demonstrate higher WS IL-15 and -18 levels and poorer periodontal status compared with individuals vaping nicotine-free e-liquids.

A limitation of the present study is that due to limited funding resources, microbiological assessment of subgingival oral biofilm for the identification of pathogenic microbes could not be performed. Moreover, based on the strict eligibility criteria, the influence of using ENDS and cigarette-smoking on immunosuppressed patients such as those with CVD and DM was not assessed. It is hypothesized that periodontal inflammatory status is poorer and WS IL-15 and -18 levels are higher in hyperglycemic individuals using ENDS and cigarettes compared with never-smokers with DM. Furthermore, treatment outcomes following periodontal therapy were not assessed as this was beyond the objectives of the current investigation. Surgical and/or non-surgical mechanical debridement are usually performed for the treatment of periodontitis [36, 37]. It is well-established that tobacco-smoking is a factor that retards periodontal wound healing following therapeutic interventions as it delays connective tissue organization and epithelial migration [36, 38]. Therefore, it is anticipated that clinical outcomes of NSPT are compromised and WS IL-15 and -18 maintain high levels had patients in groups 1 and 2 been subjected to NSPT compared with patients in Group-3. Well-designed and power-adjusted randomized controlled clinical trials are needed to test these hypotheses.

## Conclusion

Clinically, cigarette-smokers and never-smokers demonstrate similar periodontal statuses; however, WS immunoinflammatory biomarkers (IL-15 and -18) are elevated in these individuals than non-smokers.

## Data Availability

The research data and/or materials are not publicly available as the authors did not entail consents to publish this data; however, the data is available from the corresponding author upon reasonable request.
